# The Reliance on Vestibular Information During Standing Balance Control Decreases With Severity of Vestibular Dysfunction

**DOI:** 10.3389/fneur.2018.00371

**Published:** 2018-06-04

**Authors:** Joost van Kordelaar, Jantsje H. Pasma, Massimo Cenciarini, Alfred C. Schouten, Herman van der Kooij, Christoph Maurer

**Affiliations:** ^1^Department of Biomechanical Engineering, Institute for Biomedical Technology and Technical Medicine (MIRA), University of Twente, Enschede, Netherlands; ^2^Department of Biomechanical Engineering, Delft University of Technology, Delft, Netherlands; ^3^Department of Neurology, University Medical Center Freiburg, Freiburg, Germany

**Keywords:** vestibular dysfunction, gaze stabilization, vestibulo-ocular reflex, postural control, system identification

## Abstract

The vestibular system is involved in gaze stabilization and standing balance control. However, it is unclear whether vestibular dysfunction affects both processes to a similar extent. Therefore, the objective of this study was to determine how the reliance on vestibular information during standing balance control is related to gaze stabilization deficits in patients with vestibular dysfunction. Eleven patients with vestibular dysfunction and twelve healthy subjects were included. Gaze stabilization deficits were established by spontaneous nystagmus examination, caloric test, rotational chair test, and head impulse test. Standing balance control was assessed by measuring the body sway (BS) responses to continuous support surface rotations of 0.5° and 1.0° peak-to-peak while subjects had their eyes closed. A balance control model was fitted on the measured BS responses to estimate balance control parameters, including the vestibular weight, which represents the reliance on vestibular information. Using multivariate analysis of variance, balance parameters were compared between patients with vestibular dysfunction and healthy subjects. Robust regression was used to investigate correlations between gaze stabilization and the vestibular weight. Our results showed that the vestibular weight was smaller in patients with vestibular dysfunction than in healthy subjects (*F* = 7.67, *p* = 0.011). The vestibular weight during 0.5° peak-to-peak support surface rotations decreased with increasing spontaneous nystagmus eye velocity (ρ = −0.82, *p* < 0.001). In addition, the vestibular weight during 0.5° and 1.0° peak-to-peak support surface rotations decreased with increasing ocular response bias during rotational chair testing (ρ = −0.72, *p* = 0.02 and ρ = −0.67, *p* = 0.04, respectively). These findings suggest that the reliance on vestibular information during standing balance control decreases with the severity of vestibular dysfunction. We conclude that particular gaze stabilization tests may be used to predict the effect of vestibular dysfunction on standing balance control.

## Introduction

One-third of all people over 40 years old present some form of vestibular dysfunction, which often results in disturbed standing balance control and increased risk of falling (1). The vestibular organ plays an important role in balance control, although the exact mechanisms by which vestibular dysfunction affects standing balance control are still not fully understood.

Humans have two vestibular organs which are located in the inner ears and each consists of three semi-circular canals and two otoliths, which detect head angular velocity and translational acceleration, respectively (2). The vestibular organs encode the head movements in neural signals which are transmitted through the vestibular nerve to the brain (2). The brain uses the vestibular input for many purposes including standing balance control and gaze stabilization.

Gaze stabilization is mainly controlled by the vestibulo-ocular reflex (VOR). Based on semi-circular canal input, the VOR generates compensatory eye movements that are equal and opposite to head movements (3). Vestibular dysfunction leads to a reduction or absence of ocular responses to vestibular stimuli and therefore the VOR is generally examined by researchers and clinicians for the identification of vestibular dysfunction (4). For example, with the caloric test hot or cold water or air is inserted in one of the ears to evoke a vestibular response. The thermodynamics involved in the caloric test cause the fluid in the horizontal semi-circular canal to move, leading to a unilateral vestibular stimulus (5). Two other commonly used VOR tests are the rotational chair test and the head impulse test. With the rotational chair test, vestibular stimuli are evoked by horizontal whole body rotations induced by the chair (6). With the head impulse test, the head of the patient is abruptly rotated by the experimenter toward the left or right side (7, 8). In addition, spontaneous nystagmus is often examined to investigate dysfunction of the vestibular system at rest (9).

During standing balance control, the vestibular system serves, together with the visual system and the proprioceptive system, to estimate the body lean angle with respect to the environment (10). The estimated body lean angle is used by the central nervous system to control leg muscle activation, which in turn leads to the joint torques needed to maintain an upright posture (10). Due to the highly distinct neural pathways involved in the VOR and standing balance control, it is unclear how clinical VOR measures are related to the vestibular contribution in standing balance control (11). This lack of insight impedes our ability to diagnose vestibular dysfunction during standing balance control on the basis of clinical VOR measures (12).

Previous studies have shown that the contribution of each sensory and neuromuscular subsystem, including the vestibular system, can be identified with closed loop system identification techniques (10, 13–15). Compared with healthy subjects, patients with vestibular dysfunction rely less on vestibular information during standing balance control (15). In this study, we investigated the relation between the severity of vestibular dysfunction and the reliance on vestibular information during standing balance control in patients with vestibular dysfunction. Severity of vestibular dysfunction was assessed with clinical VOR measures, while reliance on vestibular information was assessed with system identification techniques (10, 16). A control group of age- and gender-matched healthy volunteers was included to evaluate to what extent balance responses and the reliance on vestibular information were different compared to patients with vestibular dysfunction.

## Materials and Methods

### Subjects

In this study, 11 patients with acute peripheral vestibular dysfunction and 12 age- and gender-matched healthy subjects were included. The data were collected at the neurology department of the University Medical Center Freiburg, Germany. All patients were admitted for acute vertigo to an outpatient section of the University Medical Center and were transferred to the neurology department for further evaluation. These patients were screened for eligibility in terms of standing capabilities and underwent routine videonystagmography of which the data were visually inspected by a neurologist (CM). Based on this visual inspection of the videonystagmography data, patients were excluded when nystagmus in the dark was absent or a peripheral vestibular deficit could not be confirmed based on the caloric test, the rotational chair test, or the head impulse test (see Procedures). Additional exclusion criteria were loss of motor function, proprioceptive impairments, and finally, inability to stand 1 min without support. All patients were included within 1 week after symptom onset and still suffered from vertigo during the experimental procedure. Healthy subjects were excluded if they suffered from any disease leading to dizziness or standing balance control disorders. To identify such a disease, healthy subjects were tested for vestibular function using Frenzel goggles on a rotational chair. The data of the healthy subjects were part of another study and were presented elsewhere (17).

Patients with vestibular dysfunction underwent routine clinical VOR examination with videonystagmography, a set of clinical balance tests and balance control measurements. Healthy subjects underwent balance control measurements only. The present study was approved by the local ethics committee and all subjects gave their informed consent prior to participation in the examinations.

### Procedures

#### Clinical Balance Tests

Balance capacity was evaluated clinically with the Berg-Balance Scale (BBS). The BBS consists of 14 items to examine postural changes from sitting to standing and *vice versa*, transfers, sitting balance, and a variety of other standing balance tasks (18, 19). For each item, a score of 0 (i.e., subject cannot do the task) to 4 (subject’s performance is normal for this task) is provided, which results in a maximum score of 56 on the BBS when no balance disabilities are detected. The BBS is mainly a measure of standing balance control and generalizes only moderately to dynamic tasks such as gait in patients with vestibular dysfunction (20). Therefore, the Timed Up and Go (TUG) scale was performed to examine mobility in patients with vestibular dysfunction (21–23). The TUG is a quick and widely used clinical performance based measure during which subjects are instructed to stand up from a chair, walk 3 m at a comfortable speed, turn around, walk back to the chair, and sit down. The time they take to complete the entire task is considered a measure of mobility.

#### Spontaneous Nystagmus

Spontaneous nystagmus was examined in the horizontal plane in complete darkness. Videonystagmography recordings were collected with the IRIS infrared eye tracker (Skalar Analytical B.V., Breda, The Netherlands) in order to obtain the eye positions, i.e., the angle of the eyes relative to the head, during these examinations. Subjects were instructed to look straight ahead. The sampling frequency during videonystagmography recordings was 200 Hz and recordings lasted 45–60 s.

#### Caloric Test

During the caloric test, subjects were seated in a chair (Nydiag 200, Interacoustics, Middelfart, Denmark) designed for videonystagmography testing, with the backrest lowered such that the trunk and head were at an angle of 30° with respect to the horizontal plane. Monothermal cold caloric test was performed with cold air of 20°C in an 8 l/min flow for 40 s. The cold air induces a downward convective current in the endolymph of the ipsilateral horizontal semi-circular canal. This current leads to cupula deflection and thereby reduces the firing rate of the afferent vestibular nerve (24). As the dynamics of the vestibular system are highly linear around the resting firing rate, a warm airflow would in principle provide the exact opposite result, i.e., an increase in neural firing rate of the vestibular nerve (25). As such, adding warm caloric test would not provide extra information about the dynamics of the vestibular system. To minimize the burden for the patient, the warm caloric test was not performed in this study. It should be noted, however, that the added value of combined cold and warm (i.e., bithermal) caloric tests is still under debate (26–29).

Directly after the application of the stimulus, lights were switched off such that the patient was in complete darkness and patients were instructed to look straight ahead. Videonystagmography recordings of horizontal eye position with a sampling frequency of 200 Hz were collected for 45 s with the IRIS infrared eye tracker and stored for further analysis. The procedure was performed for the left and right ear in random order.

#### Rotational Chair

Rotational chair testing was performed with the Nydiag 200 (Interacoustics, Middelfart, Denmark). Videonystagmography recordings of the eye position with the IRIS infrared eye tracker were collected during sinusoidal rotations of the chair in the horizontal plane, while subjects were instructed to look straight ahead. One trial was performed in which 5.5 horizontal chair oscillations at 0.2 Hz were recorded. The amplitude of the rotations was 15°. The recordings of chair position and eye position were performed with a sampling frequency of 200 Hz.

#### Head Impulse Test

Videonystagmography recordings during the head impulse test were collected with the ICS Impulse device (Otometrics, Taastrup, Denmark) while the patient was seated in a normal chair. The videonystagmography device measured the head velocity in three dimensions and horizontal eye velocity of the right eye in two dimensions (upward–downward, leftward–rightward) with a sampling frequency of 250 Hz. The present study focused on the horizontal head and eye angular velocity. The head impulse test was performed by instructing the subject to fixate the gaze on a stationary target located approximately 1 m in front of the subject on eye level. The experimenter then abruptly moved the head of the patient approximately 15° to the side with a peak velocity of approximately 200°/s. The test was repeated until at least 10 impulses to the left and 10 to the right side were recorded. Impulses direction (left/right) was presented in an alternating order.

#### Balance Control Experiment

Standing balance control was investigated with a custom-made motorized balance platform (30). This platform was used to disturb the proprioceptive information of both ankles by applying continuous support surface rotations around the ankle axis (17). For safety reasons, subjects held two ropes hanging from the ceiling with crossed arms in front of their chest, while the ropes hang loosely and not in tension so that no somatosensory spatial orientation cues could be attained from the ropes (30). The platform rotations were based on an 80 state pseudorandom ternary sequence of numbers with a time increment of 0.25 s. This sequence resulted in an angular velocity signal with a duration of 20 s. Integration of this signal resulted in an angular position signal that was used as the disturbance signal of the platform. The disturbance was repeated three times resulting in trials with a duration of 60 s.

The entire balance control experiment consisted of one quiet stance trial of 1 min and two conditions in which subjects stood on the platform with their eyes closed while the platform followed the disturbance signal with a peak-to-peak disturbance amplitude of 0.5° or 1.0°. Four disturbance trials were performed. The 0.5 peak-to-peak disturbance was provided in trial 1 and 3, and the 1.0 peak-to-peak disturbance was provided in trial 2 and 4.

The ground reaction forces were measured with a force-transducing platform (Kistler platform type 9286, Winterthur, Switzerland). In addition, the kinematics of the lower and upper body were measured with two clusters consisting of three infrared light-emitting diodes on the pelvis and shoulder (Optotrak, Northern Digital Inc., Waterloo, ON, Canada). Forces and kinematics data as well as the stimulus command signal were transferred online to a computer system (IBM compatible Pentium) *via* an analog-digital converter at a sampling rate of 100 Hz. All data were recorded with custom-made software in LabView (National Instruments, Austin, TX, USA). The body length above the ankle axis as well as the height of the pelvis and shoulder cluster relative to the ankle axis were measured. Details of the data collection have been published elsewhere (17, 30, 31).

### Data Analysis

In the analyses of all VOR measures, the sign convention used is a negative sign for head and eye movements toward the left and a positive sign for head and eye movements toward the right.

#### Spontaneous Nystagmus

To investigate the VOR during spontaneous nystagmus, a time segment of 10 s was selected during which the subject did not blink or change the direction of gaze. Subsequently, the eye position signal of the right eye in the horizontal plane was differentiated to obtain the eye angular velocity signal. The left eye position signal was discarded in all analyses of the VOR as VOR responses typically result in synchronous eye movements (32). The fast phases of the VOR were removed from the velocity signal (25) using a custom-made routine using Matlab (Matlab Version 2013a, The MathWorks, Natick, MA, USA) as described in Supplementary Materials A.

The spontaneous nystagmus was quantified as the median slow phase eye velocity during spontaneous nystagmus (*MV*_SN_). The raw data and the result of the spontaneous nystagmus for one patient are presented in Supplementary Figure B.1 of Supplementary Materials B.

#### Caloric Test

To detect the eye velocity in the slow phases, the same routine as described for spontaneous nystagmus was conducted for the caloric test. The median slow phase eye velocities after stimulation with cold air in the left (*MV*_CL_) and right ear (*MV*_CR_) were extracted from 10 s time intervals after stimulation during which the subject did not blink or change the direction of gaze. Jongkee’s formula was used to calculate the vestibular asymmetry index as established during the caloric test (*AI*_CAL_):

(1) $$AI_{\text{CAL}}=\frac{MV_{\text{CR}}+MV_{\text{CL}}}{MV_{\text{CR}}-MV_{\text{CL}}}.$$

Note that in general *MV*_CR_ is positive, whereas *MV*_CL_ is negative. The numerator of Eq. 1 is then defined as the difference between *MV*_CR_ and *MV*_CL_, whereas the denominator is defined as the sum of these values. A value of 0 means that the response to caloric test is normal, i.e., symmetric. A value of −1 indicates absence of a right response, whereas a value of 1 indicates absence of a left response. The raw data and the result of the caloric test for one patient are presented in Supplementary Figure B.1 of Supplementary Materials B.

#### Rotational Chair

The chair and the eye position signals obtained during the rotational chair test were filtered using a fourth-order recursive low-pass Butterworth filter with a cutoff frequency of 10 and 40 Hz, respectively. The first and the last half chair rotation cycle and corresponding eye movements were discarded to avoid artifacts induced by the acceleration from and deceleration to the chair rest position. From the remaining 4.5 cycles, the chair position and eye position signal were differentiated to obtain the eye velocity signal. Fast phases of the VOR were removed with the routine as described in Supplementary Materials A. Remnants of eye blinks that were not correctly identified by the fast phase removal routine were manually removed from the data.

Regression analysis was used to estimate the ocular response gain (*G*_ROT_) and the ocular response bias (*B*_ROT_) during the rotational chair test as indicated by, respectively, the slope and intercept of the linear fit between chair velocity and slow phase eye velocity (25). *G*_ROT_ is a measure of the strength of the ocular response to the chair rotations, where an ocular response gain of −1 indicates an ocular response velocity that is exactly opposite to the chair rotation velocity. An ocular response gain smaller or larger than −1 indicates an ocular response velocity that is, respectively, larger or smaller than the chair rotation velocity. For example, a gain of −2 would indicate a response velocity that is opposite to and twice as large as the chair rotation velocity, whereas a gain of 0 would indicate no response at all. *B*_ROT_ is a measure of the ocular response velocity offset, which is the ocular response velocity when the chair rotation velocity is 0°/s (25). Raw data and the regression analysis of the rotational chair data for one patient are presented in Supplementary Figure B.2 of Supplementary Materials B.

#### Head Impulse Test

The head and eye velocity signals were filtered using a fourth-order recursive low-pass Butterworth filter with a cutoff frequency of 60 Hz. Head impulses were included for analysis when the peak velocity was larger than 130°/s. Head impulses and corresponding eye velocity signals were excluded when the head velocity signal was not smooth and contained more than one peak or when the eye velocity signal contained eye blinks represented by peaks that exceeded 2.5 times the peak head velocity. The start of the head impulse was defined by the time instant at which the head velocity exceeded the threshold of 20°/s. Likewise, the end of the head impulse was defined as the time instant at which the head velocity fell below this threshold. When the eye velocity signal was not stationary before the start of a head impulse and exceeded the threshold within the 0.06 s before the head impulse, the head impulse and the corresponding eye velocity signal were excluded.

Correction saccades in the eye velocity signal, represented by peaks in the eye velocity signal were removed with the fast phase removal routine described in Supplementary Materials A. Linear interpolation was used to fill the gaps in the eye velocity signal. To quantify the VOR during each head impulse, the area under the head velocity signal and the eye velocity signal was calculated by numerical integration of each signal. The ocular response gain for each head impulse was obtained by dividing the area under the eye velocity signal by the area under the head velocity signal. The median ocular response gain was calculated for left (*G*_L_) and right (*G*_R_) head impulses separately. An ocular response gain of 0 indicates no response at all, whereas an ocular response gain of −1 indicates a response that is exactly opposite to the head rotation velocity. Finally, the vestibular asymmetry index as established during the head impulse test (*AI*_HIT_) was calculated:

(2) $$AI_{\text{HIT}}=\frac{G_{\text{R}}-G_{\text{L}}}{G_{\text{R}}+G_{\text{L}}}.$$

An *AI*_HIT_ value of 0 means that the response to the head impulse test is normal, i.e., symmetric. An *AI*_HIT_ value of −1 indicates absence of a right response, whereas an *AI*_HIT_ value of 1 indicates absence of a left response. Raw head impulse data for one subject are presented in Supplementary Figure B.3 of Supplementary Materials B.

#### Balance Control Experiment

The body weight of the subject was estimated by dividing the mean ground reaction force during the quiet stance trial by the gravitational acceleration. Leg and trunk angles with respect to the vertical were calculated from the displacements of the pelvis and shoulder clusters and their respective heights. Based on these leg and trunk angles, the position of the center of mass and the body sway (BS) angle were calculated (33). To eliminate transient effects, the first disturbance cycle of 20 s was removed from each trial, resulting in two cycles per trial. As each condition was performed twice, the signals of the disturbance command and the BS angle were segmented into four data blocks of 20 s.

For each condition, the response of the BS to the disturbance was obtained by estimating the sensitivity functions. To this end, the data blocks of the disturbance command signal and the BS angle were transformed to the frequency domain using discrete Fourier transform. Subsequently, the frequency coefficients were averaged across data blocks, yielding a mean disturbance signal and a mean BS response signal in the frequency domain. The power spectral density of the disturbance signal as well as the cross-spectral density between the disturbance signal and the BS were calculated for the excited frequencies. For each condition and each subject, the sensitivity functions were estimated using the indirect approach by dividing the cross spectral density by the power spectral density (34). The resulting sensitivity function provides the magnitude and phase, which describe the response of the balance control system at each excited frequency. The magnitude indicates how much the disturbance signal is amplified in the BS signal, whereas the phase indicates the phase lag of the BS relative to the disturbance signal.

**Figure 1:**
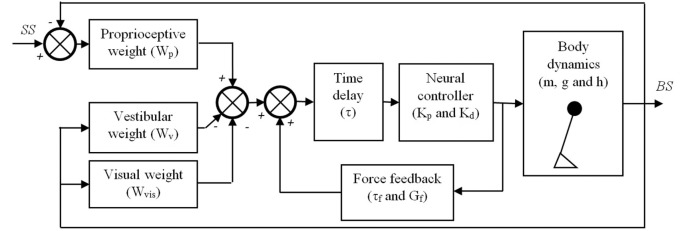
Balance control model that was used in this study. The body is represented by a single inverted pendulum of which the dynamics depend on its (point) mass (*m*), the height (*h*) of this mass above the ankle joints, and the gravitational constant (*g*). The body sway (BS) is sensed by three sensory systems, i.e., the proprioceptive system, the vestibular system, and the visual system. It is assumed that the contribution of each system to the sensory feedback signal depends on its reliability and is given by their weights, *W*_p_, *W*_v_, and *W*_vis_, respectively. The sensed BS is fed into a neural controller with a time delay (τ). The neural controller is assumed to be a proportional-derivative (PD) controller represented by a stiffness (*K*_p_) and damping (*K*_d_), which generates the torques needed to maintain an upright posture of the inverted pendulum. Finally, a positive force feedback loop of which the dynamics are given by a force feedback gain (*G*_f_) and a force feedback time constant (τ_f_) is assumed to assist in the detection of the BS, primarily on the lower frequencies.

For each subject and for each condition, the parameters of a balance control model (Figure 1) were estimated by fitting the mathematical sensitivity function of the balance control model onto the experimental sensitivity function. The mass and the height of the center of mass in the balance control model were derived from the body weight and body length and were used as fixed parameters (33). The moment of inertia was calculated using the mass and length of the center of mass (33). The model was fitted on the sensitivity function for each condition and each subject using a nonlinear least-square fit (Matlab function: lsqnonlin.m) by minimizing the sum squared error (*E*) between the experimental sensitivity function and the mathematical transfer function of the balance control model for the excited frequencies (*f*) (16):

(3) $$\begin{array}{l} \varepsilon (f)=\sqrt{\frac{\gamma_{\text{SS,BS}}^{2}(f)}{f}}\cdot \left|\text{log}\left(\frac{S_{\text{exp}}(f)}{S_{\text{fit}}(f)}\right)\right|\\ E=\frac{1}{N}\varepsilon (f){\;}^{T}\varepsilon (f), \end{array}$$

where $\gamma_{SS,BS}^{2}$ is the coherence between the support surface rotations and BS, and *S*_exp_ and *S*_fit_ are the experimental and fitted sensitivity functions, respectively. *N* is the number of excited frequencies.

This procedure resulted in the following estimated parameters for each condition: (1) proprioceptive weight (*W*_p_) indicating the reliance on proprioceptive feedback, (2) reflexive stiffness (*K*_p_) indicating the stiffness induced by the neural controller, (3) reflexive damping (*K*_d_) indicating the damping induced by the neural controller, (4) force time constant (τ_f_), (5) force feedback gain (*G*_f_), and (6) a lumped time delay (τ), as also shown in Figure 1. As subjects had their eyes closed during the balance control experiments, no visual information was available and therefore the visual weight was 0. Therefore, the vestibular weight, indicating the reliance on vestibular information, was defined by: *W*_v_ = 1 − *W*_p_ (10). *K*_p_ and *K*_d_ depend on the gravitational torque which is determined by the gravitational acceleration (*g*) as well as the mass (*m*) and the estimated height (*h*) of the inverted pendulum that represents the subject’s body. Therefore, to allow comparison between subjects *K*_p_ and *K*_d_ were normalized by dividing by the gravitational torque (i.e., m*g*h).

To investigate the goodness of the model fits, the goodness of fit (GOF) in the frequency domain was calculated as follows:

(4) $$\text{GOF}=\left[1-\frac{\sum_{k=1}^{N}{\left|S_{\text{fit}}(f_{k})-S_{\text{exp}}(f_{k})\right|}^{2}}{\sum_{k=1}^{N}{\left|S_{fit}(f_{k})\right|}^{2}}\right]*100\%.$$

#### Statistics

To assess differences in the balance control model parameters between conditions and groups a multivariate analysis of variance (MANOVA) was performed, with the model parameters as dependent variables and group and condition as a between and within factor, respectively. In case, the MANOVA showed a significant effect of group or condition, *post hoc* tests were performed using separate two-way mixed analyses of variance (ANOVAs) for each model parameter, with group as a between subject factor and condition as a within subject factor.

To assess the relation between *W*_v_ and each VOR measure, robust regression with a bisquare weighting function and a tuning constant of 4.685 was used (35). The same robust regression analysis was used to assess correlations between VOR measures in case multiple VOR measures were related to *W*_v_. Level of significance was set two-sided at *p* < 0.05 for all statistical tests. MANOVA was conducted using SPSS version 20 (IBM, Armonk, NY, USA). Robust regression was performed with Matlab.

## Results

There were no significant differences between patients with vestibular dysfunction and healthy subjects regarding age (*t* = 0.12, *p* = 0.90), body weight (*t* = 1.27, *p* = 0.22), and body length (*t* = 1.15, *p* = 0.26). Patients with vestibular dysfunction had a median BBS score of 53 (interquartile range: 50.5–55) out of the maximum score of 56 on this test. The median outcome of the TUG scale was 9.4 s (interquartile range: 7.3–15.4 s). Four patients with vestibular dysfunction had a TUG scale larger than 11.1 s which indicates an increased risk of falling (36). Table 1 lists the demographics and outcomes of the BBS and TUG scale, the outcomes of the VOR tests, and the proprioceptive weights for each condition for the 11 patients and 12 healthy subjects who were included in this study.

**Table 1:** Subject demographics and outcomes.^a^

Outcome	Patients with vestibular dysfunction	Healthy subjects
Number	11	12
Age (years)	61.4 ± 13.4	60.8 ± 10.7
Gender (female/male)	5/6	5/7
Body length (m)	1.73 ± 0.10	1.68 ± 0.11
Body weight (kg)	83.44 ± 11.51	76.27 ± 15.08
*W*_0.5_ (–)	0.12 ± 0.09	0.24 ± 0.08
*W*_1.0_ (–)	0.21 ± 0.14	0.31 ± 0.07
BBS^b^ (–)	53.0 [50.5–55.0]	–
TUG^b^ (s)	9.4 [7.3–15.4]	–
MV_SN_ (°/s)	4.5 ± 4.1	–
AI_CAL_ (–)	0.61 ± 0.38	–
*G*_ROT_ (–)	−0.39 ± 0.22	–
*B*_ROT_ (°/s)	3.1 ± 3.5	–
AI_HIT_ (–)	0.20 ± 0.14	–

*^a^Variables are presented as mean with SD, unless indicated otherwise*.

*^b^Median [interquartile range]*.

*AI_CAL_, absolute value of the asymmetry index for the caloric test; AI_HIT_, absolute value of the asymmetry index for the head impulse test; B_ROT_, absolute value of the eye velocity bias during the rotational chair test; BBS, Berg-Balance Scale; G_ROT_, ocular response gain during the rotational chair test; MV_SN_, absolute value of the mean eye velocity during spontaneous nystagmus examination; TUG, Timed Up and Go scale; W_0.5_, vestibular weight for the 0.5° peak-to-peak condition; W_1.0_, vestibular weight for the 1.0° peak-to-peak condition*.

### Standing Balance Control in Patients With Vestibular Dysfunction and Healthy Subjects

**Figure 2:**
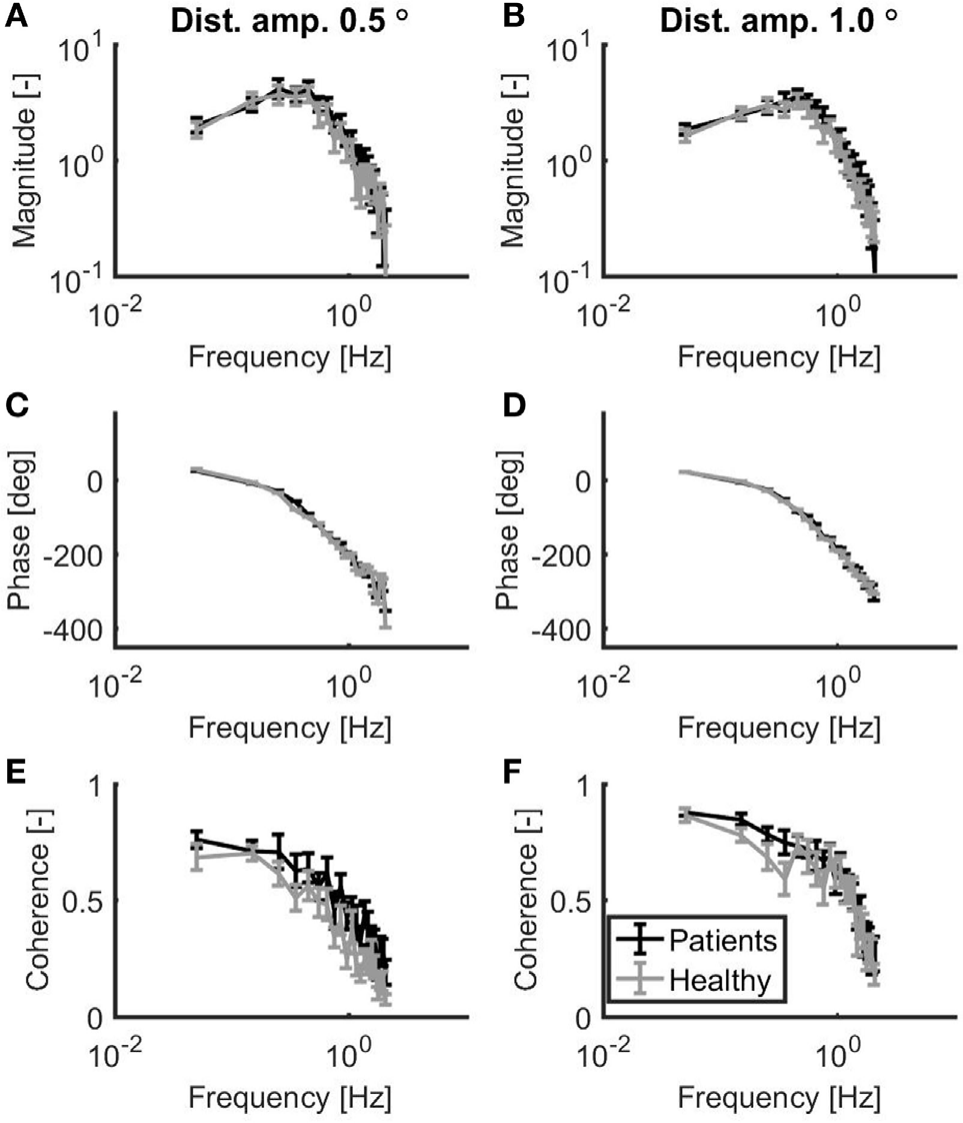
Mean magnitude **(A,B)** and mean phase **(C,D)** of the sensitivity functions as well as the mean coherence **(E,F)** for a peak-to-peak disturbance amplitude of 0.5° **(A,C,E)** and 1.0° **(B,D,F)**. Patients with vestibular dysfunction and healthy subjects are represented by black and gray lines, respectively. Error bars represent SE.

As shown in Figure 2, patients with vestibular dysfunction showed a slightly larger magnitude of the response to the support surface rotation, especially for the frequencies larger than 0.3 Hz. The goodness of the balance control model fits was assessed by the GOF metric. The mean (±SD) GOF for the patients with vestibular dysfunction in the 0.5° and 1.0° peak-to-peak condition was 80 (±16) and 88 (±7)%, respectively. Likewise, the GOF values for the healthy control subjects in the 0.5° and 1.0° peak-to-peak condition were 79 (±13) and 88 (±9)%, respectively.

**Figure 3:**
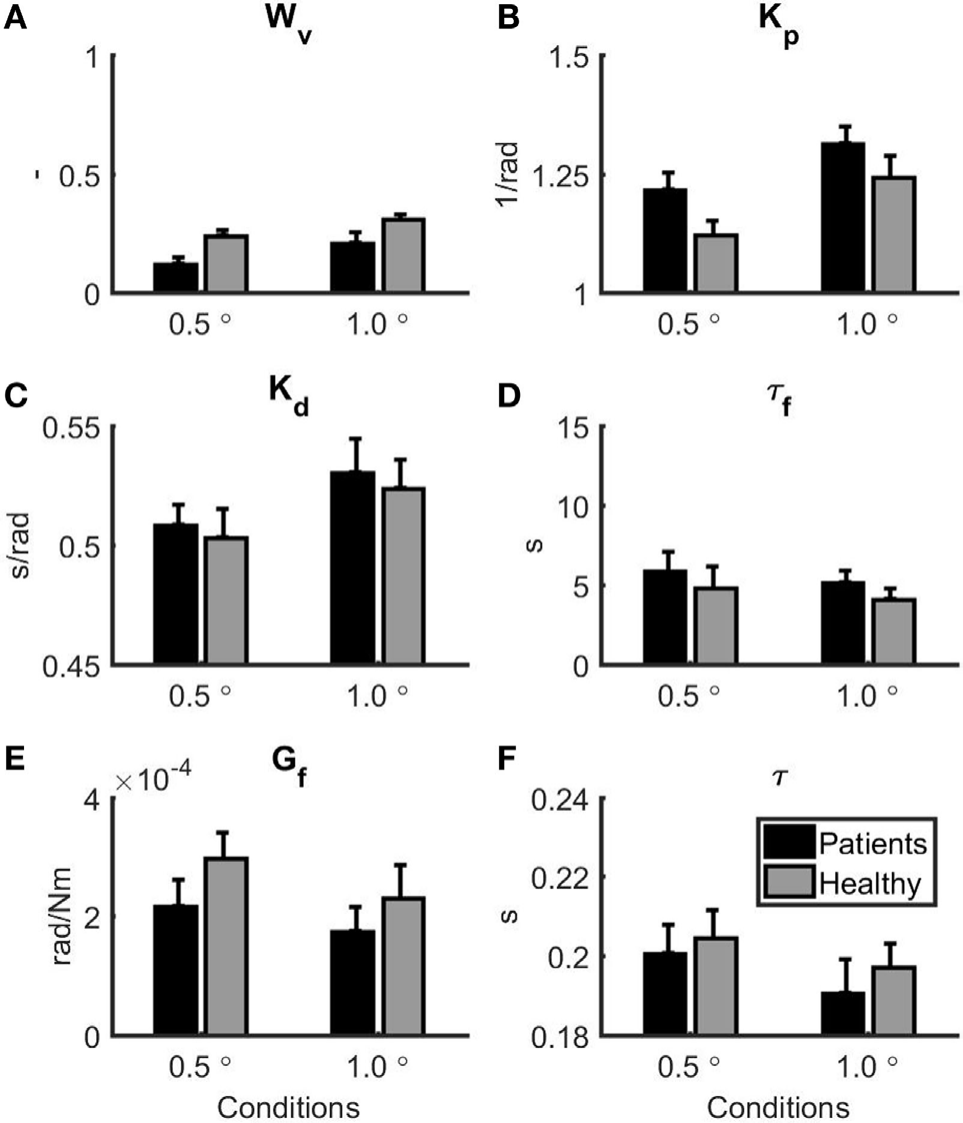
Parameter estimates for the patients with vestibular dysfunction (black) and healthy subjects (gray). 0.5° represents the 0.5° peak-to-peak condition and 1.0° represents the 1.0° peak-to-peak condition. Error bars represent SE. **(A)** Vestibular weight, **(B)** reflexive stiffness, **(C)** reflexive damping, **(D)** force feedback time constant, **(E)** force feedback gain, and **(F)** time delay.

Figure 3 shows the mean and SE for the balance model parameters for each group. Based on a MANOVA with Pillai’s trace as a test statistic, there was a significant main effect of condition (*F* = 13.46, *p* < 0.001) on the collection of model parameters, whereas there was no main effect of group on the collection of model parameters (*F* = 1.88, *p* = 0.146). The interaction between group and condition was also not significant (*F* = 0.16, *p* = 0.985). Univariate two-way mixed ANOVAs showed several significant differences between groups and conditions. *W*_v_ was significantly lower in patients with vestibular dysfunction as compared to healthy subjects (*F* = 7.67, *p* = 0.011). In addition, *W*_v_ was significantly lower in the 1.0° peak-to-peak as compared to the 0.5° peak-to-peak condition (*F* = 22.00, *p* < 0.001). *K*_p_ was significantly lower in the 0.5° peak-to-peak as compared to the 1.0° peak-to-peak condition (*F* = 11.31, *p* = 0.003). *K*_p_ was also higher in the group with patients with vestibular dysfunction as compared to healthy subjects. This group effect reached borderline significance (*F* = 4.15, *p* = 0.055). For *K*_d_ and τ, there were main effects of condition indicating that *K*_d_ was lower (*F* = 17.29, *p* < 0.001) and τ was higher (*F* = 7.24, *p* = 0.014) in the 0.5° peak-to-peak as compared to the 1.0° peak-to-peak condition. No further main or interaction effects were found.

### Relation Between VOR Measures and Vestibular Weight

**Figure 4:**
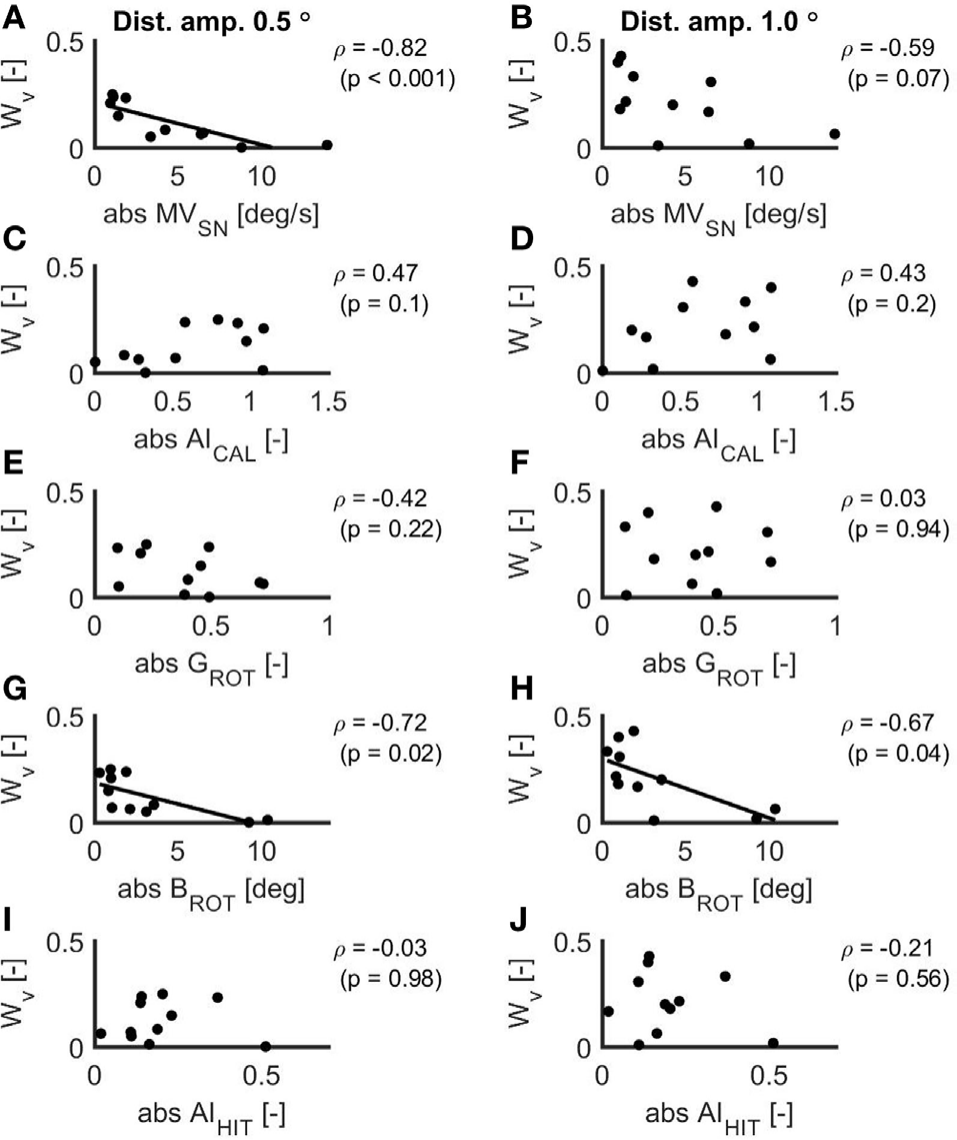
Scatterplots of the relations between the absolute values of the mean eye velocity during spontaneous nystagmus examination **(A,B)**, the asymmetry index as established with the caloric test **(C,D)**, the ocular response gain during the rotational chair test **(E,F)**, the ocular response bias during the rotational chair test **(G,H)** and the asymmetry index as established with the head impulse test **(I,J)** and the vestibular weight during a peak-to-peak disturbance amplitude of 0.5° **(A,C,E,G,I)** and 1.0° **(B,D,F,H,J)**. In case of a significant relation, the linear fit is included in the scatterplot.

The scatterplots for the relations between the VOR measures and *W*_v_ for the two conditions in the balance experiment are displayed in Figure 4. *MV*_SN_ (ρ = −0.82, *p* < 0.001) and *B*_ROT_ (ρ = −0.72, *p* = 0.02) correlated significantly with *W*_v_ for the 0.5° peak-to-peak condition. For these relations, the linear fit is included in the scatterplot. In addition, *B*_ROT_ correlated significantly with *W*_v_ for the 1.0° peak-to-peak condition (ρ = −0.67, *p* = 0.04). There was a strong relationship between *B*_ROT_ and *MV*_SN_ (ρ = 0.92, *p* < 0.001).

## Discussion

In this study, we assessed the relation between the severity of vestibular dysfunction assessed with clinical gaze stabilization tests and the reliance on vestibular information during standing balance control during forward–backward support surface rotations in patients with vestibular dysfunction. The main result was that the reliance on vestibular information during standing balance control decreased with increasing ocular response bias measured with the rotational chair test and the mean eye velocity measured during spontaneous nystagmus examination. This finding suggests that the severity of vestibular dysfunction determines how much patients rely on vestibular information during standing balance control. To our knowledge, this study is the first to establish such a link between vestibular dysfunction and reliance on vestibular information during balance control.

### Standing Balance Control in Patients With Vestibular Dysfunction and Healthy Subjects

The scores on the BBS were relatively high in our patient group, indicating that the ability to maintain standing balance was close to normal. However, the Timed Up and Go scale revealed balance disabilities during gait in a subgroup of the patients in this study. Four patients with vestibular dysfunction exceeded a previously reported threshold of 11.1 s suggesting that these patients had an increased risk of falling (36).

By using system identification techniques, we also revealed a subtle but significant difference in standing balance control between patients with vestibular dysfunction and healthy subjects. The vestibular weight in patients with vestibular dysfunction was significantly smaller than in age- and gender-matched healthy subjects, which implies that patients with vestibular dysfunction rely less on vestibular information during standing balance control as compared to healthy subjects. This finding is in line with a recent study that reported similar values for the vestibular weight in patients with vestibular dysfunction and healthy subjects during sideward support surface disturbances (15). In addition, reflexive stiffness was (insignificantly) higher in patients with vestibular dysfunction as compared to healthy subjects, suggesting that patients with vestibular dysfunction actively increase the stiffness of their ankle joints. Such an increase in ankle stiffness is probably the result of reflex modulations at supra-spinal level (37) and could be a beneficial strategy to increase the stability of balance when it is accompanied with an increase in reflexive damping (38). Although not significant, the present study also suggested increased reflexive damping in patients with vestibular dysfunction as compared to healthy subjects. These findings suggest that our patients with vestibular dysfunction may have changed their neural response in order to compensate for reduced vestibular function during stance (39).

Both groups adapted their control of balance to the disturbance amplitude. In particular, the vestibular weight increased with increasing disturbance amplitude of the support surface rotation, which is line with previous studies (10, 15). Apparently, patients with vestibular dysfunction relied less on vestibular information, however, they preserved the ability to scale their reliance on vestibular information to the disturbance amplitude. In addition, in both groups, reflexive stiffness and damping increased with increasing disturbance amplitudes, which may reflect a strategy to stabilize balance control when external disturbances increase (38). Finally, the estimated neural time delay was similar to the time delay found in other studies (10, 15, 38, 40, 41). Also, the decrease in time delay with increasing support surface disturbance is in line with previous studies (10, 15).

### Relation Between Vestibular Dysfunction During Gaze Stabilization and Vestibular Weight During Balance Control

The vestibular weight was related to the spontaneous nystagmus eye velocity and the ocular response bias during the rotational chair test. Both VOR measures quantify the eye velocity when the head velocity is zero and the similarity between these VOR measures is also reflected by the strong and significant correlation between them. These measures reflect the eye velocity that is induced by an imbalance in resting discharge firing rate between the intact and dysfunctional vestibular sides (42). This response is typically present in patients with acute vestibular dysfunction and commonly disappears as a result of vestibular compensation processes when patients progress toward the chronic phase (43). As a larger eye velocity when head velocity is zero indicates a more severe vestibular dysfunction the identified relation with the vestibular weight indicates that the reliance on vestibular information during stance decreases with increasing severity of vestibular dysfunction. A previous study already showed that spontaneous nystagmus levels were related to BS excursions during quiet stance on foam with eyes closed (12). This study extends on this finding indicating a direct relationship between vestibular dysfunction and vestibular contributions to balance control.

It is difficult to deduce why particularly the zero head velocity VOR measures were related to the vestibular weight whereas the other three VOR measures included in this study were not. However, we can list a number of possible explanations.

First, the subtle disturbance amplitudes used in the balance control experiment resulted in relatively small BS amplitudes and low sway velocities. Due to the low sway velocities, the vestibular input during the balance control experiments is likely much lower than the vestibular input during the caloric test, the rotational chair test, and the head impulse test. This low vestibular input during the balance control test may therefore explain why particularly the eye velocity during zero head velocity correlated with the vestibular weight.

Second, vestibular dysfunction generally manifests itself in one of the two organs (44) and therefore the severity of the vestibular dysfunction is best captured by measures that quantify the function of one organ relative to the other. The ocular response gain measured with the rotational chair test essentially quantifies the function of the entire vestibular system and this poor distinction between affected and non-affected side may explain why the ocular response gain measured with the rotational chair test was not related to the vestibular weight during standing balance control.

Third, the vestibular stimulus during the caloric test is generated indirectly through an airflow in the ears. Due to the thermal variation, the density of the endolymph changes, particularly in the horizontal semi-circular canal, leading to formation of convection currents and deflection of the cupula (24). However, apart from the temperature of the airflow, anatomical properties of the temporal bone, such as its density, may also influence the strength of the ocular response (45) and may therefore weaken the relation with the vestibular weight. This confounding effect may therefore explain why we did not find any correlations between asymmetric caloric responses and the reliance on vestibular information during standing balance control. In addition, some studies suggest that warm and cold (i.e., bithermal) caloric testing may increase the reliability and diagnostic accuracy in comparison with monothermal caloric testing (26, 46). Therefore, the reduced accuracy of the monothermal cold caloric test in this study may have obscured a correlation between caloric test results and the reliance on vestibular information during standing balance control.

These three possible reasons can each individually or in conjunction have played a role in weakening the relation with the vestibular weight during standing balance control.

### Methodological Considerations and Future Directions

The number of participants who were included in this study was relatively small. Nonetheless, a significant relation between the severity of vestibular dysfunction and the reliance on vestibular information during standing balance control could be established. However, with regard to several differences in standing balance control parameters between patients with vestibular dysfunction and healthy subjects statistical significance was not achieved. For example, the increased reflexive stiffness in patients with vestibular dysfunction as compared to healthy subjects was close to significant (*p* = 0.055). These differences in standing balance control may probably be significantly revealed when future studies increase the sample size.

With respect to the relation between vestibular function and standing balance control, we deliberately related standing balance control parameters to VOR tests that are commonly used in clinical practice. However, these VOR tests mainly test the function of the horizontal semi-circular canal, whereas for balance control the central nervous system may rely mainly on information from the anterior and posterior canal as well as the otoliths (11). In addition, as patients progress from the acute to the chronic phase after symptom onset vestibular compensation processes in the brain occur, leading to a reduction of vestibular symptoms including spontaneous nystagmus (43, 47). The found relation between spontaneous nystagmus and the reliance on vestibular information during standing balance control is therefore specifically applicable to patients with acute vestibular dysfunction.

Future studies should include examinations of all semi-circular canals and otoliths in order to establish which anatomical parts of the vestibular system are mostly related to vestibular dysfunction during standing balance control. In addition, future studies should assess VOR and standing balance control in a longitudinal manner in order to investigate how the relation between the severity of vestibular dysfunction and the reliance on vestibular information during standing balance control changes as a function of time after symptom onset (12, 48).

## Conclusion

Our data showed a correlation between gaze stabilization deficits and the reliance on vestibular information during standing balance control in patients with vestibular dysfunction. Based on this finding, we conclude that the reliance on vestibular information during standing balance control decreases with the severity of vestibular dysfunction. In particular, the eye velocity when the head angular velocity is zero was related to the reliance on vestibular information during standing balance control, which suggests that this gaze stabilization measure may be used to predict vestibular dysfunction during standing balance control in clinical practice. Future studies should measure gaze stabilization longitudinally in order to investigate how dysfunction of the semi-circular canals and otoliths relates to vestibular reliance during standing balance control and how these relations change over time after symptom onset.

## Ethics Statement

The protocol for this study was carried out in the Neurology department of the University of Freiburg in accordance with the ethics committee of the University of Freiburg. All subjects were verbally informed about all procedures prior to participation in the examinations. As all tests reported for patients with vestibular dysfunction were part of routine examinations that were primarily conducted for clinical and diagnostic purposes, patients provided verbal but not written informed consent. The data of the healthy subjects were part of another study. All healthy subjects gave written informed consent prior to participation in the experiments.

## Author Contributions

CM and JK designed the study. JK collected the data and drafted the manuscript. JP, MC, and JK analyzed the data. All authors contributed to the interpretation of the data and approved the final version of the manuscript for submission. JP, MC, AS, HK, and CM critically revised the manuscript.

## Conflict of Interest Statement

The authors declare that the research was conducted in the absence of any commercial or financial relationships that could be construed as a potential conflict of interest.

## Abbreviations

BBS, Berg-Balance Scale; BS, body sway; (M)ANOVA, (multivariate) analysis of variance; SE, standard error; TUG, Timed Up and Go scale; VOR, vestibulo-ocular reflex.
