# Supplementary material for: The Reliance on Vestibular Information During Standing Balance Control Decreases With Severity of Vestibular Dysfunction
Source: Front Neurol. 2018 Jun 4;9:371. doi: 10.3389/fneur.2018.00371 (PMC5994722; doi:10.3389/fneur.2018.00371)
Supplement: Supplementary file 1 [file data_sheet_1.PDF]

Supplementary Material A

The reliance on vestibular information during standing balance control decreases with severity of vestibular dysfunction

Joost van Kordelaar*, Jantsje H. Pasma, Massimo Cenciarini, Alfred C. Schouten, Herman van der Kooij, Christoph Maurer

*** Correspondence:** Joost van Kordelaar: j.vankordelaar@gmail.com

1 Introduction

To remove the fast phases of vestibulo-ocular reflex (VOR) responses from eye velocity signals, the fast phase removal routine was composed in Matlab (Matlab Version 2013a, The MathWorks, Natick, Massachusetts, USA). Fast phases are characterized by large spikes in eye velocity signals as shown in Supplementary Figure A.1 for rotational chair data. In general, fast phases exhibit an acceleration phase directly followed by a deceleration phase. Both phases have an amplitude which is much larger than the accelerations involved in slow phase VOR responses and they occur within a small time window. These two aspects of fast phases are used to identify these fast phases in the eye acceleration signal.

2 Description of the fast phase removal routine

The routine follows a stepwise approach to remove the fast phases, characterized by spikes in the eye velocity signal. First, it differentiates the eye velocity signal (EV) to obtain the eye acceleration signal (EA). Example data are shown in Supplementary Figure A.1. Second, it searches for all epochs during which EA is larger or falls below an acceleration threshold (T_{acc}), which is set by the user. This step is shown in Supplementary Figure A.2. Third, it identifies peaks in the eye velocity signal by searching for pairs of two consecutive epochs in which the acceleration is positive in one epoch and negative in the other. Importantly, a fast phase segment is short by definition. Therefore, the peaks in the eye velocity signal are identified only when the two epochs in the acceleration signal are shorter than a maximal time (T_{max}) set by the user. Fourth, a fast phase is then defined as the time interval between the first data point and the last data point of the pair of epochs. Fifth, a short interval is added before (T_{pre}) and after (T_{post}) each time interval as the first and last samples of a fast phase were typically not identified. The lengths of these intervals are set by the user. Lastly, samples that were identified as a fast phase were set to ‘Not-a-Number’ (NaN). The eye velocity signal from which the fast phases are removed is shown in Supplementary Figure A.3.

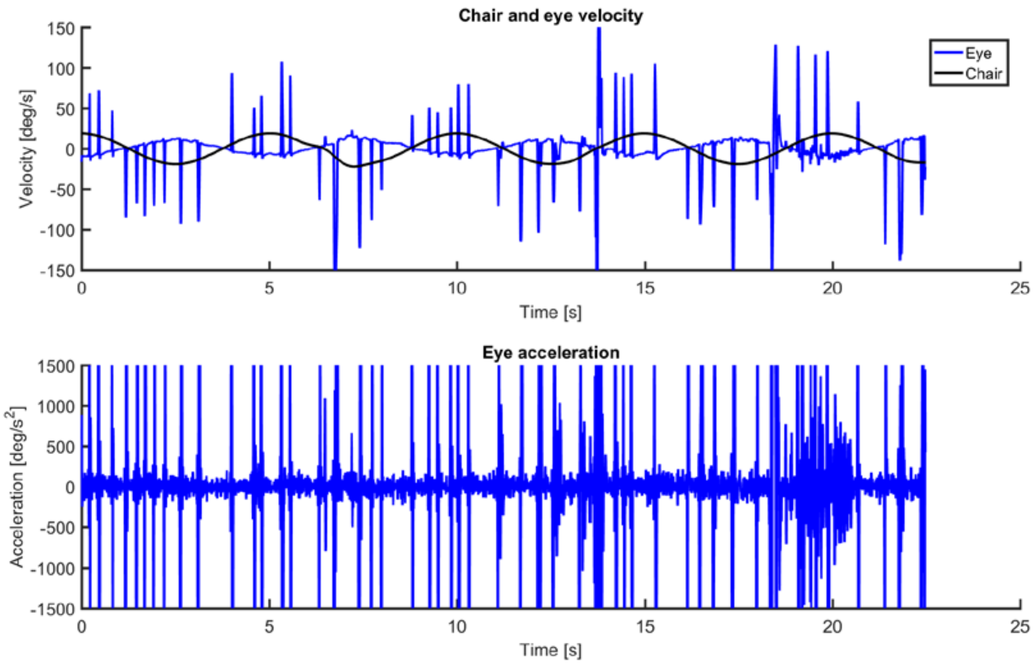

Supplementary Figure A.1. Raw data from a rotational chair recording in a representative patient. Upper panel: chair and eye velocity. Lower panel: Eye acceleration. The large spikes in the eye acceleration signal are induced by the fast phases of the VOR.

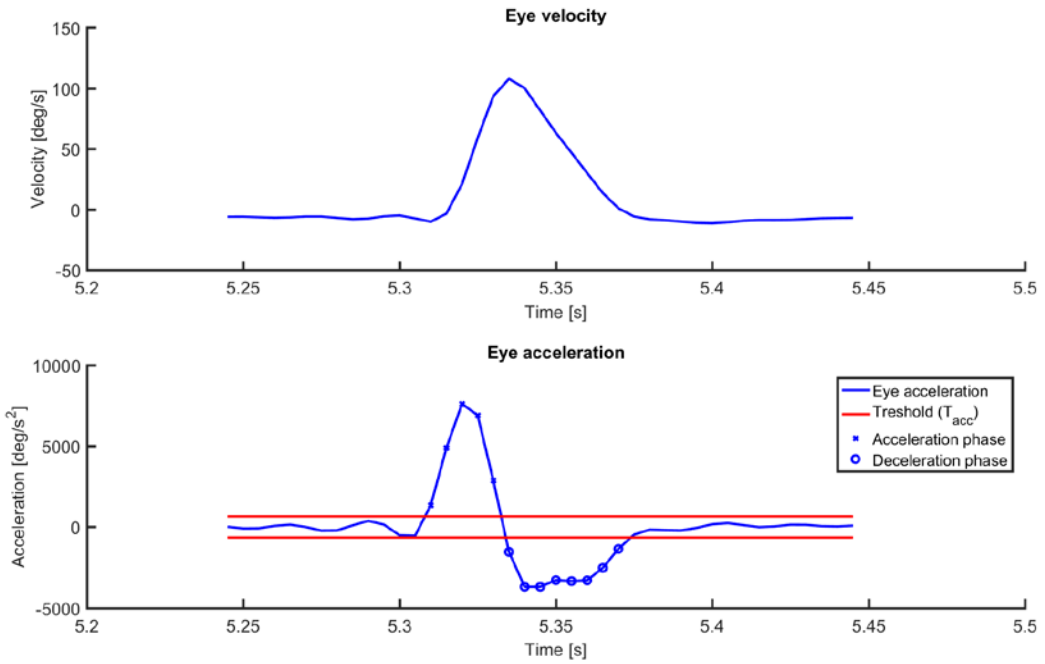

Supplementary Figure A.2. Selection of rotational chair data shown in Figure A.1. Upper panel: Eye velocity. Lower panel: Eye acceleration with acceleration threshold (T_{acc}). T_{acc} was set to 650 deg/s^2. Data points that were identified as part of acceleration and deceleration phase are marked with 'x' and 'o', respectively. Note that the duration of the acceleration phase and the deceleration phase was smaller than the T_{max} which was in this case set to 0.135 s.

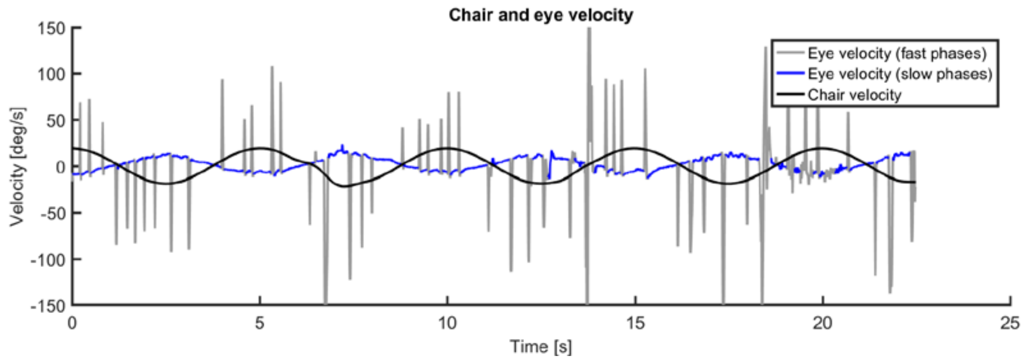

Supplementary Figure A.3. Raw eye velocity data (grey), the eye velocity data without the fast phases (blue) and the chair velocity data (black).
