# Supplementary material for: The Reliance on Vestibular Information During Standing Balance Control Decreases With Severity of Vestibular Dysfunction
Source: Front Neurol. 2018 Jun 4;9:371. doi: 10.3389/fneur.2018.00371 (PMC5994722; doi:10.3389/fneur.2018.00371)
Supplement: Supplementary file 2 [file data_sheet_2.PDF]

Supplementary Material B

The reliance on vestibular information during standing balance control decreases with severity of vestibular dysfunction

Joost van Kordelaar*, Jantsje H. Pasma, Massimo Cenciarini, Alfred C. Schouten, Herman van der Kooij, Christoph Maurer

*** Correspondence:** Joost van Kordelaar: j.vankordelaar@gmail.com

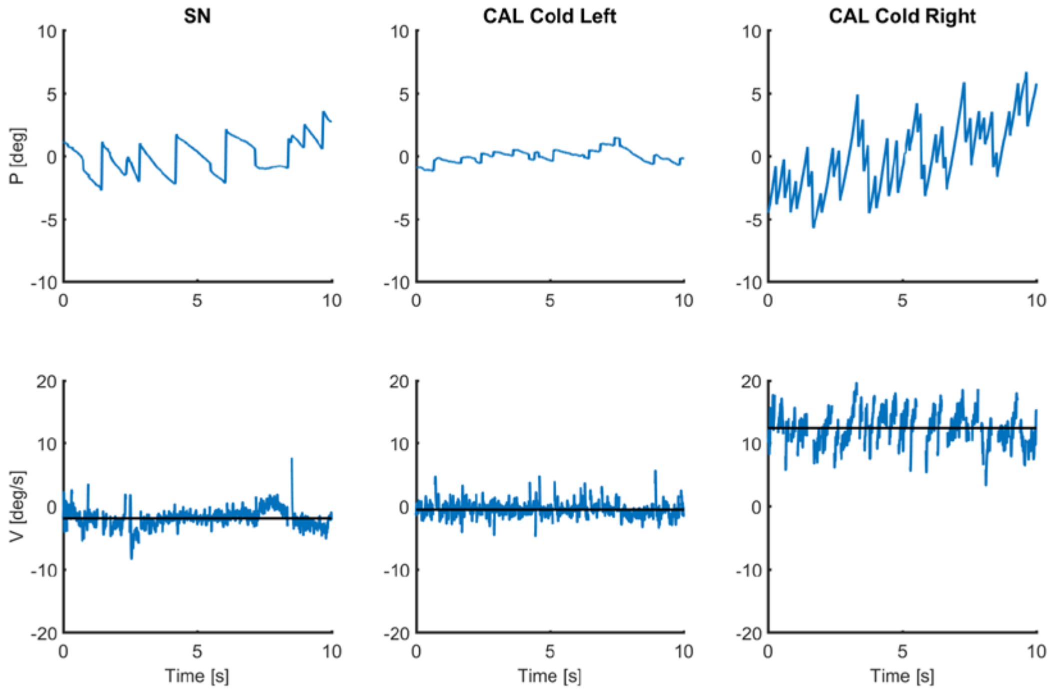

Supplementary Figure B.1 Raw eye position signals (upper panels) and eye velocity signals with fast phases removed (lower panels) for spontaneous nystagmus (left), caloric left cold (middle) and caloric right cold (right) for one patient. Negative/positive values indicate movements to the left/right. The black line in the lower panels indicates the median slow phase eye velocity. This patients showed a small spontaneous nystagmus (slow phase) to the left. Compared to the right ear, the response during cold caloric stimulation of the left ear was minimal, indicating a left vestibular deficit in this patient, which agrees with the small spontaneous nystagmus to the left.

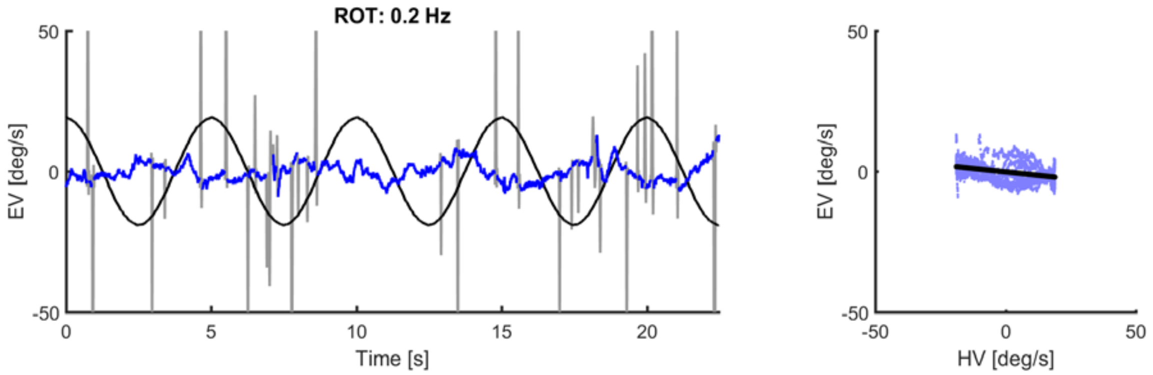

Supplementary Figure B.2 Analysis of rotational chair data. Left panel: Raw eye velocity (shaded) and eye velocity data with fast phases removed (blue) for one patient at 0.2 Hz chair rotations. The harmonic chair rotation is represented by the black line. Right panel: corresponding regression line (black) based on slow phase eye velocity vs head velocity (blue).

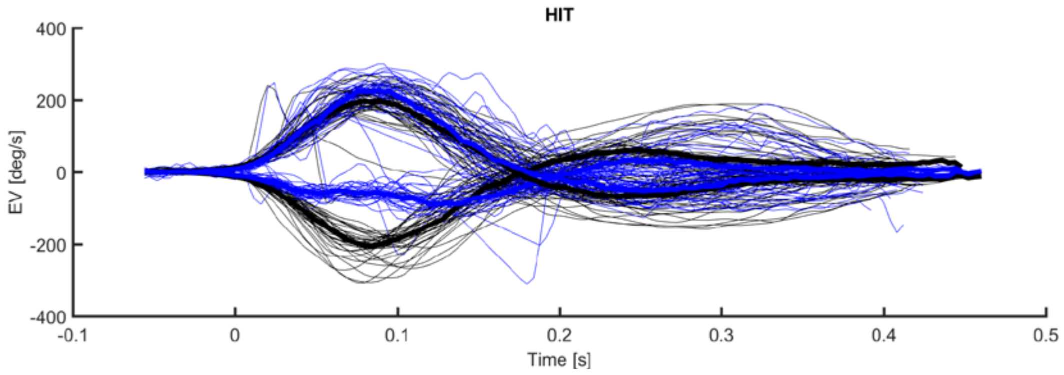

Supplementary Figure B.3 Raw head impulse data for one patient. Thin black lines represent head movement velocity during single head impulses. Thin blue line represents the eye movement responses to these head impulses. Fast phases are removed using linear interpolation. Positive/negative lines correspond to rightward/leftward head impulses and corresponding responses. This patient clearly head a weaker response to leftward head impulses, indicating a left vestibular deficit and corresponding to the spontaneous nystagmus to the left and absence of a response to a left cold caloric stimulus (see Supplementary Figure B.1).
